# Strategy towards tailored donor tissue-specific pancreatic islet isolation

**DOI:** 10.1371/journal.pone.0216136

**Published:** 2019-05-10

**Authors:** Yuki Miyazaki, Kazutaka Murayama, Ibrahim Fathi, Takehiro Imura, Youhei Yamagata, Kimiko Watanabe, Hiroshi Maeda, Akiko Inagaki, Yasuhiro Igarashi, Shigehito Miyagi, Hiroki Shima, Kazuhiko Igarashi, Takashi Kamei, Michiaki Unno, Masafumi Goto

**Affiliations:** 1 Department of Surgery, Tohoku University Graduate School of Medicine, Sendai, Miyagi, Japan; 2 Graduate School of Biomedical Engineering, Tohoku University, Sendai, Miyagi, Japan; 3 Division of Transplantation and Regenerative Medicine, Tohoku University Graduate School of Medicine, Sendai, Miyagi, Japan; 4 Department of Applied Biological Chemistry, Graduate School of Agricultural Science, Tokyo University of Agriculture and Technology, Fuchu, Tokyo, Japan; 5 New Industry Creation Hatchery Center, Tohoku University, Sendai, Miyagi, Japan; 6 Department of Biochemistry, Tohoku University Graduate School of Medicine, Sendai, Miyagi, Japan; University of Minnesota Medical Center, UNITED STATES

## Abstract

**Background:**

Optimizing the collagenase G (ColG):collagenase H (ColH) ratio is a key strategy for achieving tailored donor-tissue specific islet isolation. Collagen V (Col V) and collagen III (Col III) are crucial target matrices of ColG and ColH, respectively. We herein investigated the relevance between the expression of target matrices in pancreatic tissues and influence of ColG:ColH ratio on islet isolation outcome.

**Methods:**

Islet isolation was performed in Lewis and SD rats using different ColG:ColH ratios (5:1, 1:1 and 1:5; n = 7/group). The composition of Col III and Col V was examined using immunohistochemical staining, real-time polymerase chain reaction (PCR), Western blotting and mass spectrometry. Chain types in collagen I (Col I) were also assessed using mass spectrometry.

**Results:**

No beneficial effects were observed by increasing the ColG amount, irrespective of the rat strain. In contrast, the islet yield in Lewis rats was considerably increased by high amounts of ColH but decreased in SD rats, suggesting that Lewis pancreas contains more Col III than SD pancreas. Neither immunohistochemical nor real-time PCR showed correlation with isolation outcome. However, Western blotting revealed that Lewis contained considerably higher amount of Col III than SD (p = 0.10). Likewise, Col-I(α1)/Col-III(α1) and Col-I(α2)/Col-III(α1) were significantly lower in Lewis than in SD rats (p = 0.007, respectively). Furthermore, the isolation outcome was considerably correlated with the composition of homotrimeric Col I.

**Conclusions:**

The Col III expression and the composition of homotrimeric Col I in pancreatic tissues determined using mass analyses appeared useful for optimizing the ColG:ColH ratio in islet isolation.

## Introduction

Although pancreatic islet transplantation is a promising and safe therapy for type 1 diabetic patients[1, 2], many issues remain to be solved. For example, pancreata from two or more donors are required for one diabetic patient to achieve insulin independence in many cases, despite improvements in human pancreatic islet isolation procedures over the past three decades [3–6]. Furthermore, the successful islet isolation rate has increased through appropriate donor selection, but the successful islet isolation rate of whole donor pancreata is still poor [7, 8]. A more efficient islet isolation procedure may increase the islet yield from one donor pancreas and solve such problems, helping relieve the organ shortage issue.

An important factor associated with the outcome of islet isolation is the tissue dissociation enzyme [4, 9, 10]. A donor-specific, individualized, islet isolation protocol can theoretically be established if highly purified components of tissue dissociation enzyme were prepared to target the extracellular matrix of each enzyme component.

Enzymes currently used for pancreatic islet isolation include collagenase, neutral protease and other unknown components. Collagenases, produced by *Clostridium histolyticum* (*C*. *histolyticum*), are the main components of the islet isolation enzyme combination. These collagenases are consisted of six different collagenases, and they are divided into two groups; collagenase G (ColG) and collagenase H (ColH) [11, 12]. ColG (corresponding to Class I collagenases) has a high activity toward native collagen and a moderate activity toward 2-furanacryloyl-L-leucylglycyl-L-prolyl-L-alanine (FALGPA), whereas ColH (corresponding to Class II collagenases) has a moderate activity toward collagen and a high activity toward FALGPA [11]. We have successfully produced tryptic-like activity (TLA)-free enzyme preparations of highly purified recombinant collagenase of each subtype to examine the roles of ColG and ColH in islet isolation [13–15], given Brandhorst et al.’s finding that some unknown proteases other than collagenases and neutral proteases, which have a high TLA, might affect pancreas dissociation [16]. Using these novel collagenases, we clearly showed that ColH is crucial for rat pancreatic islet isolation [13].

We also reported that collagen III (Col III) is a key target matrix of ColH using highly purified recombinant collagenases [13]. Given that Col III is most intensely expressed in the exocrine tissues, with almost no expression in the peri-insular region [13], islet isolation is likely initiated by fragmentation of the pancreatic tissues by ColH mainly via the degradation of Col III. We recently demonstrated that collagen V (Col V) is only digested by ColG and not by ColH, and that Col V is a key substrate for ColG [17]. Given these findings, we hypothesized that the semi-quantitative assessment of Col III and V in pancreatic tissues might aid in successful islet isolation by directing the optimization of the ColG and ColH levels.

To achieve a tailor-made type of donor tissue specific islet isolation procedure, we investigated the relationship between the islet isolation outcome and different rat strains using variable ColG:ColH ratios. We also evaluated the Col III and Col V expression in pancreatic tissues of different rat strains using immunohistochemical (IHC) staining, real-time polymerase chain reaction (PCR), Western blotting and mass spectrometry and correlated the findings with the influence of the ColG:ColH ratio on the islet isolation outcome.

We believe that the findings of the present study may open the way to establish the most suitable combination of tissue dissociation enzyme components for any type of donors, especially for young donors and highly fibrotic pancreata that have been well known to cause poor outcome of islet isolation.

## Materials and methods

### Animals

Rat pancreata were obtained from 9-week-old inbred Lewis rats and 8-week-old closed colony SD rats (Japan SLC, Inc., Shizuoka, JAPAN). All animals used in this present study were treated according to the *Guide for the Care and Use of Laboratory Animals* published by the National Institutes of Health. The protocol was approved by the ethics committee for animal experiments and related activities of Tohoku University (approved protocol ID: 2016 Medical-Animal-197). All surgical procedures were performed under inhalation anesthesia using isoflurane, and every effort was made to reduce suffering. All animals were sacrificed with deep anesthesia and bleeding caused by cutting inferior vena cava.

### Enzyme preparation

Recombinant ColG and ColH (Meiji Seika Pharma Co., Ltd., Tokyo, Japan) were used with thermolysin (TL) as a neutral protease (Peptide Institution, Inc., Osaka, Japan) to prepare the enzyme blends. The activity of TL (0.065 mg) was adjusted to that of the crude collagenase from *C*. *histolyticum* (Sigma collagenase type V; Sigma-Aldrich, St. Louis, MO, USA) using Azocasein (Sigma-Aldrich Japan, Tokyo, Japan). The activities of ColG and ColH were calculated using FALGPA and Azocoll (Calbiochem, Merck Millipore, Darmstadt, Germany). We calculated the protein amount of ColG and ColH when their activities in the enzyme blends were equal to those of Sigma collagenase type V. Each rat strain was divided into three groups regarding the ratio of ColG to ColH (5:1, 1:1 and 1:5 groups). The ratio was calculated using the protein amount of each collagenase, and the total protein amount of ColG and ColH was adjusted 20% (1.49 mg) to that calculated from Sigma collagenase Type V (5:1 group: ColG 1.242 mg, ColH 0.248 mg; 1:1 group: ColG 0.745 mg, ColH 0.745 mg; 1:5 group: ColG 0.248 mg, ColH 1.242 mg). All enzyme blends were diluted in Hanks’ Balanced Salt Solution (HBSS).

### Islet isolation

Rat islet isolation was performed as previously described [18]. After cannulating the bile duct, 10 mL of cold HBSS containing the enzyme blends was injected followed by the removal of the pancreas. After digestion at 37°C for 14 min, purification by a density-gradient centrifugation was performed using a Histopaque-1119 (Sigma Diagnostics, St. Louis, MO, USA) and Lymphoprep (Axis-Shield PoC AS, Oslo, Norway). The islet count was determined as islet equivalents (IEQs) with diphenylthiocarbazone (Wako Pure Chemical Industries, Ltd., Osaka, Japan) staining. The isolated islets were cultured in Roswell Park Memorial Institute-1640 medium containing 5.5 mmol/L glucose (Thermo Fisher Scientific, Inc., Waltham, MA, USA) and 10% fetal bovine serum (Equitech-Bio, Inc., Kerrville, TX, USA) at 37°C in 5% CO_2_ and humidified air.

### Immunohistochemical staining of rat pancreatic tissues

Rat pancreatic tissues were cut into small sections, embedded into Tissue-TK O.C.T. Compound (Sakura Finetek USA, Inc., Torrance, CA, USA) and frozen with liquid nitrogen after fat tissues, vessels and lymph nodes were removed. We obtained 8-μm slices from the frozen pancreatic tissue and placed them in 4% paraformaldehyde for 10 min at room temperature. The slides were pretreated with 5 mg/mL of pepsin (Dako, Agilent Technologies, Inc., Santa Clara, CA, USA) at 37°C for 10 min for antigen retrieval, and with 10% bovine serum albumin (Sigma-Aldrich) for 20 min at room temperature to block non-specific antibody reaction. Antibodies against Col III (Anti-Collagen III antibody; Abcam PLC, Cambridge, UK) and Col V (Anti-Collagen V antibody; Abcam) were used as primary antibodies for immunolabeling at 1:80 and 1:100 dilutions in phosphate-buffered saline (PBS) respectively. As a secondary antibody, EnVision System-HRP Labelled Polymer Anti-Rabbit (Dako, Agilent Technologies) was used, followed by localization of antibody bindings with diaminobenzidine (Trevigen, Inc., Gaithersburg, MD, USA). Slides were washed twice with PBS between each process. In all slices, the percentage of the stained area was calculated using the Image J software program, ver. 1.51n (National Institute of Health, Bethesda, MD, USA) [19].

### Col3a1 and Col5a1 mRNA in pancreatic tissues

Pancreatic tissues were obtained from each rat strain (n = 5, respectively) under anesthesia and immediately placed in RNAlater (QIAGEN, Hilden, Germany) after removing fat tissues, vessels and lymph nodes. Samples were then cut into small sections and frozen in liquid nitrogen. Total RNA was extracted using the RNeasy Mini Kit (QIAGEN) according to the manufacturer’s protocol. The RNA extraction quality was evaluated by the ratio of absorbance at 260 nm/280 nm. First-strand complementary DNA (cDNA) was synthesized from 2.5 μg of total RNA using the Transcriptor First Strand cDNA Synthesis Kit (Roche Diagnostics, Indianapolis, IN, USA). The cDNA was amplified by PCR using a rat Col3a1 primer and a probe (5’ to 3’ direction forward primer: ATG AGC TTT GTG CAA TGT GG, 5’ to 3’ direction reverse primer: CTA GAC TCA TAG GAC TGA CCA AG, 5’ to 3’ direction probe: FAM-CCT GGT TTC TTC TCA CCC TGC TTC ACC-TAMRA) (Nihon Gene Research Laboratories Inc., Sendai, Japan), rat Col5a1 primer and a probe (5’ to 3’ direction forward primer: TGT GCC ACT CGA CGA TCT, 5’ to 3’ direction reverse primer: GTC CTC AGG AAA GTC AGA CTC A, 5’ to 3’ direction probe: FAM-CCA AAG AGC CGG ATG TTG CCT ACC-TAMRA) (Nihon Gene Research Laboratories), and rat glyceraldehyde-3-phosphate dehydrogenase (GAPDH) primer and a probe (Nihon Gene Research Laboratories) as housekeeping gene with a LightCycler FastStart DNA Master HybProbe (Roche Diagnostics) and LightCycler 2.0. To perform the PCR for Col3a1 and Col5a1, an initial denaturation step of 10 minutes at 95°C was followed by 45 cycles of 10 seconds at 95°C, annealing and extension of 30 seconds at 60°C. For GAPDH, an initial denaturation step of 10 minutes at 95°C was followed by 40 cycles of 10 seconds at 95°C, annealing and extension of 60 seconds at 60°C.

### Western blotting of Col III in pancreatic tissues

Pancreatic tissues were obtained from each rat strain (n = 5, respectively) under anesthesia and frozen in liquid nitrogen after removing fat tissues, vessels and lymph nodes. A small section of pancreas was obtained from several random locations and placed in PBS (7.5 μl of liquid per one milligram of tissue) containing 5 mM AEBSF (Nacalai Tesque, Inc., Kyoto, Japan), 4 μM Aprotinin (Nacalai Tesque), 75 μM E-64 (Nacalai Tesque), 100 μM Leupeptin (Nacalai Tesque), 250 μM Bestatin (Nacalai Tesque), 50 μM Pepstatin A (Nacalai Tesque), 10 mM EDTA (Nacalai Tesque), 1 mM TLCK (Nacalai Tesque), and 1 mM TPCK (Wako Pure Chemical Industries). The samples were homogenized using POLYTRON PT 10–35 (Kinematica AG, Luzern, Switzerland) at 10,000 rpm for 1 min repeated 5 times each. After homogenization, the suspension was mixed with one quarter of 1 M hydrochloric acid (Nacalai Tesque) and left on ice for 60 minutes while stirring with vortex every 10 minutes. After centrifuging at 4°C and 15,000 *g* for 10 min, the supernatant was collected. The concentration of protein in supernatants were measured using a BCA Protein Assay kit (Thermo Fisher Scientific, Inc.). Protein contained in the supernatant was precipitated by adding trichloroacetic acid, and after centrifuging at 4°C and 15,000 *g* for 20 min, the precipitate was suspended in SDS buffer, containing 1.2% SDS (Nacalai Tesque), 60 mM Tris-HCl buffer (pH 6.8), 12% glycerol (Nacalai Tesque), 0.04% BPB (Nacalai Tesque), and 10% 2-mercaptoethanol (Nacalai Tesque), at 5 mg/ml or 1 mg/ml and boiled for 5 minutes. The 20 μl of 5 mg/ml and 10 μl of 1 mg/ml sample solutions were separated by sodium dodecyl sulfate-polyacrylamide gel electrophoresis (SDS-PAGE) in the col III analysis and the β-actin analysis, respectively. SDS-PAGE was performed using a 7.5% polyacrylamide slab gel for the col III analysis and 12.5% for the β-actin analysis, respectively. The separated proteins were transferred onto polyvinylidene fluoride membranes (Bio-Rad Laboratories, Inc., Hercules, CA, USA) using a Trans-Blot SD semi-dry transfer cell (Bio-Rad Laboratories). The membrane was reacted with Anti-Collagen III antibody (catalog No. ab7778, Abcam) or Anti-β-actin antibody (catalog No. ab8227, Abcam) as the primary antibody. The secondary antibody was alkaline phosphatase-conjugated goat anti-rabbit IgG (catalog No. ab6722, Abcam) and signals were detected using nitro blue tetrazolium (Nacalai Tesque) and 5-bromo-4-chloro-3-indolyl phosphate. The scanned and captured bands were analyzed using ImageJ 1.52a software developed at the National Institutes of Health [19].

### Western blotting of Col V in pancreatic tissues

Pancreatic tissues were obtained from each rat strain (n = 5, respectively) under anesthesia and frozen in liquid nitrogen after removing fat tissues, vessels and lymph nodes. A small section of pancreas was obtained from several random locations and placed in 1000 μL of PBS containing 5 mmol/L EDTA (Nacalai Tesque), 10 mmol/L benzamidine (Merck Millipore) and 0.1 mg/mL soybean trypsin inhibitor (Sigma-Aldrich), and 10 μL of 99.5% ethyl alcohol containing 100 mmol/L phenylmethylsulfonyl fluoride (PMSF) (Sigma-Aldrich). The samples were homogenized using PT1300E (Kinematica AG) and VCX-130 (Sonics and Materials, Inc., Newtown, CT, USA) for 1 min each. After centrifuging at 4°C and 3,400 rpm for 10 min, the supernatant was collected and stored at -80°C. The concentration of protein in supernatants were measured using a BCA Protein Assay kit (Thermo Fisher Scientific) and adjusted to 1mg/mL with NOVEX Tris-Glycine SDS Sample Buffer (Thermo Fisher Scientific). The samples were separated by SDS-PAGE using Novex 4–12% Tris-Glycine Mini Gels, WedgeWell format (Thermo Fisher Scientific) and Novex Tris-Glycine SDS Running Buffer (Thermo Fisher Scientific). The separated samples were transferred to PVDF Pre-cut Blotting Membranes (Thermo Fisher Scientific), which were blocked by ECL Prime Blocking Reagent (GE Healthcare UK Ltd., Amersham, UK). Thereafter, the membranes were incubated with Anti-Collagen V (catalog No. ab114072, Abcam) and anti-β-actin antibody (catalog No. ab8227, Abcam) at 4°C overnight, and Goat Anti-Rabbit IgG H&L (HRP) (catalog No. ab205718, Abcam) was used as second antibody for 60 min at room temperature. The bands were visualized by Amersham ECL Prime (GE Healthcare Ltd., UK) and analyzed using a Molecular Imager VersaDoc MP 5000 System (Bio-Rad Laboratories).

### In vitro digestion of pancreatic tissues

Pancreatic tissues were prepared for each rat strain (Lewis and SD). Tissues (n = 8 for each rat strain) were divided into small pieces (typically 40–60 mg) on ice. The tissue pieces were then homogenized with Micro Smash MS-100R (TOMY MEDICO, Tokyo, Japan) with stainless beads (φ 3mm). The homogenized solutions were subsequently applied to the CNMCS compartmental protein extraction kit (Millipore, Billerica, MA, USA) to enrich collagens [20, 21]. The samples were digested using S-Trap: Rapid Universal MS Sample Prep (PROTIFI, Huntington, NY, USA) with trypsin according to the manufacturer’s instructions.

### Liquid chromatography with tandem mass spectrometry of collagens

The digested samples were applied to MS analyses. The liquid chromatography with tandem MS (LC-MS) system comprised a PAL HTC-xt autosampler (AMR Inc., Tokyo, Japan) and Ultimate 3000 pump (Thermo Fisher Scientific) connected to a C18 tip column (10 cm length and 75 μm inner diameter, Nikkyo Technos Co., Ltd. Tokyo, Japan), and Orbitrap LTQ-Velos (Thermo Fisher Scientific). Raw data were processed with the Proteome Discoverer version 1.4.1.14 (Thermo Fisher Scientific) using the standard workflow. A database search was performed using the Mascot search engine (version 2.6.0, Matrix Science, London, UK) against SwissProt. Quantitative evaluations were conducted using an emPAI index [22].

### Statistical analyses

All values were expressed as the mean ± standard deviation and analyzed using the JMP Pro software program, ver. 13.1.0 (SAS Institute Inc., Cary, NC, USA). Data from two groups were compared using Mann-Whitney U test. Data from three groups were compared using Kruskal-Wallis test and Steel-Dwass test as a post hoc test. A value of *p* < 0.05 was considered to indicate statistical significance.

## Results

### Effects of the collagenase subtype (ColG:ColH) ratio on the islet yield by rat strains

**Fig 1:**
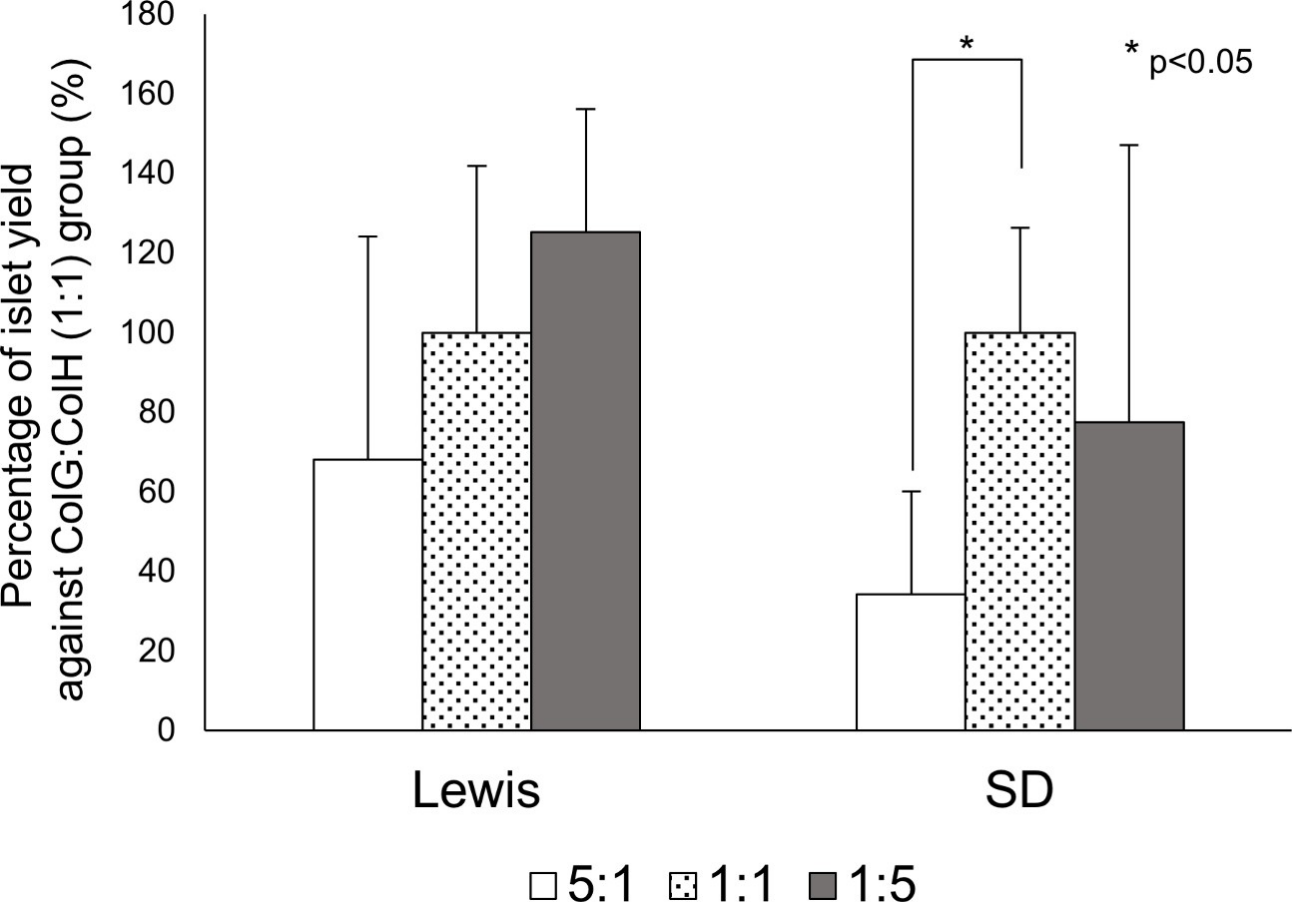
Effects of the ColG:ColH ratio on the islet yield for each rat strain. The islet yields of the 5:1 (white) and 1:5 (gray) groups are presented as the percentage when the yield of the 1:1 group (dotted) was considered 100% in each strain.

To clarify the effects of the collagenase subtype ratio on the islet yield, the exact same lot and amount of TL was used for all experimental groups. The islet yield was not increased by a high amount of ColG in either strain (Fig 1). In contrast, the yield was considerably increased by a high amount of ColH in the Lewis rats (ColG:ColH (1:1): 1799.2 ± 753.4 IEQs, ColG:ColH (1:5): 2254.9 ± 555.4 IEQs, p = 0.09, n = 7), but tended to decrease in the SD rats (ColG:ColH (1:1): 944.6 ± 248.5 IEQs, ColG:ColH (1:5): 732.2 ± 658.2 IEQs, n = 7) (Fig 1). In all groups of both rat strains, the function of isolated islets was not affected in FDA/PI analysis (data are not shown).

### Immunohistochemical staining of rat pancreatic tissues

**Fig 2:**
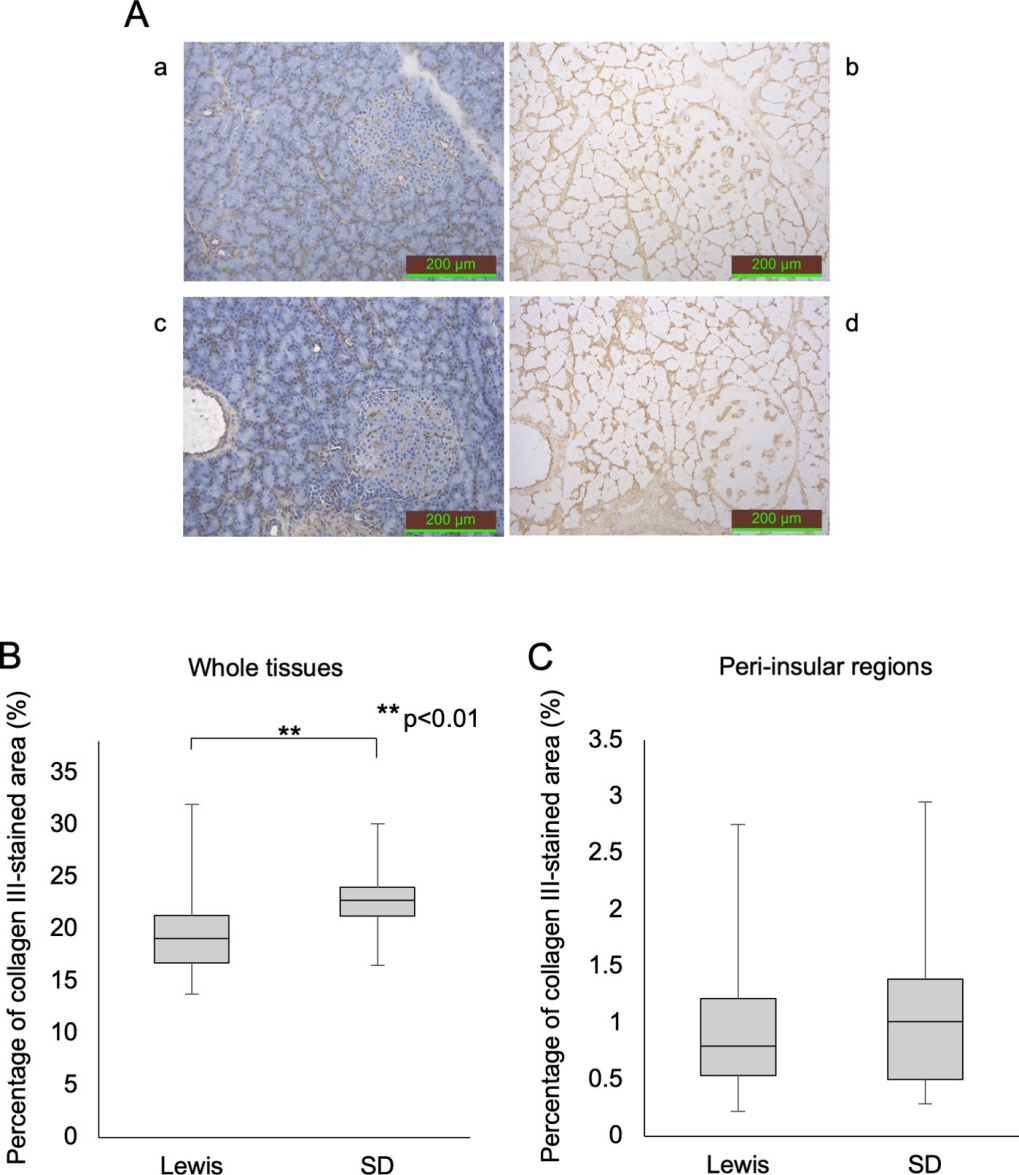
Anti-collagen III staining of the pancreatic tissues in each rat strain. (A) Anti-collagen III staining (a and b: Lewis rat; c and d: SD rat). The stained area was calculated using pictures without hematoxylin staining (b, d). (B) The percentage of the over-all anti-collagen III-stained area. (C) The percentage of the anti-collagen III-stained area in the peri-insular region only.

**Fig 3:**
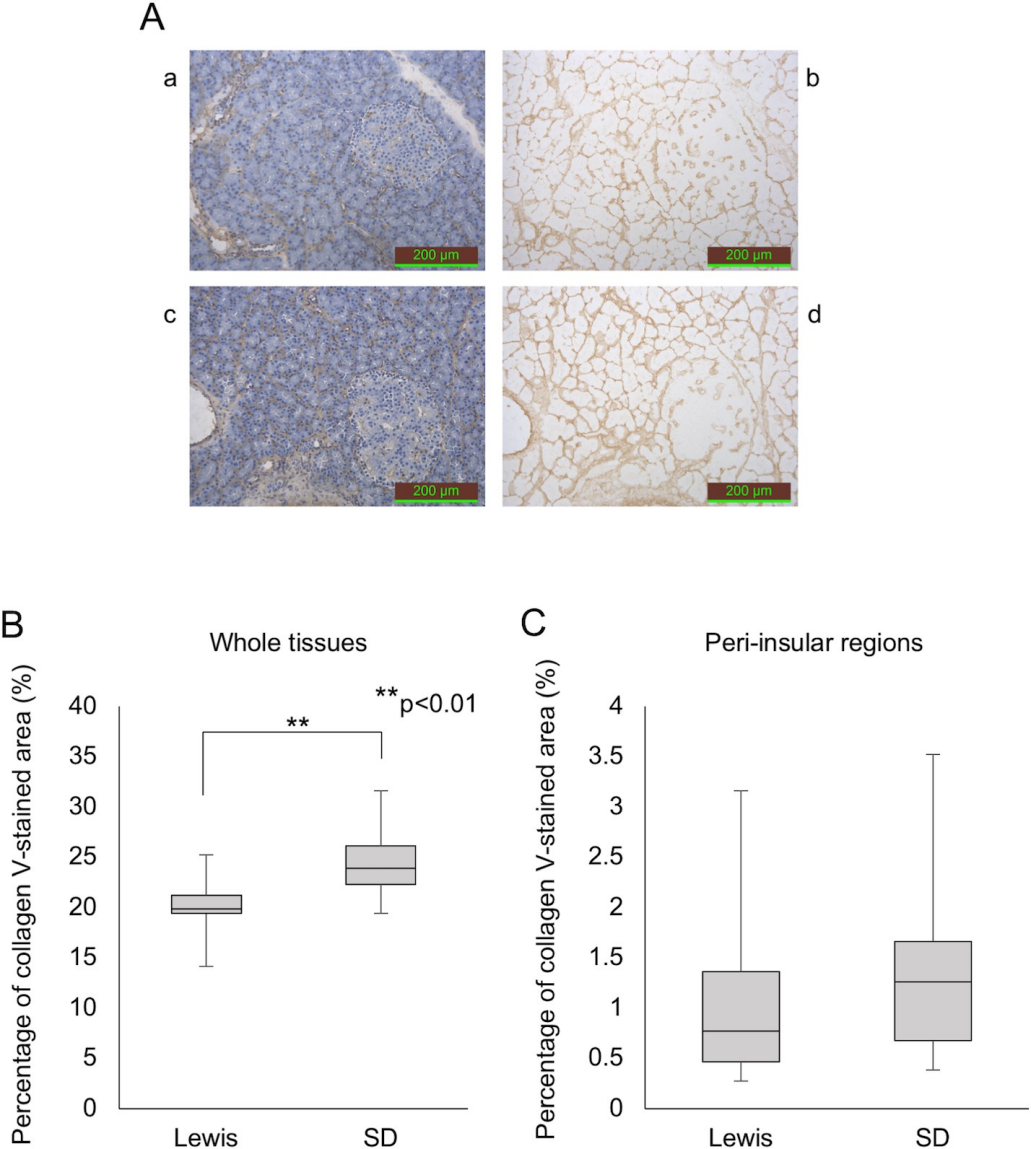
Anti-collagen V staining of the pancreatic tissues in each rat strain. (A) Anti-collagen V staining (a and b: Lewis rat; c and d: SD rat). The stained area was calculated using pictures without hematoxylin staining (b, d). (B) The percentage of the overall anti-collagen V-stained area. (C) The percentage of the anti-collagen V-stained area in the peri-insular region only.

The percentage of Col III- and V-stained areas in the whole tissues of SD rats was significantly higher than in those of Lewis rats (collagen III: 22.8% ± 3.1% vs. 20.0% ± 4.6%, p<0.001, collagen V: 24.3% ± 2.9% vs. 20.0% ± 1.9%, p<0.001) (Figs 2B and 3B). No marked differences were observed in the Col III and V staining in the peri-insular regions between the strains (Figs 2C and 3C).

### mRNA expression of Col3a1 and Col5a1 in rat pancreatic tissues

**Fig 4:**
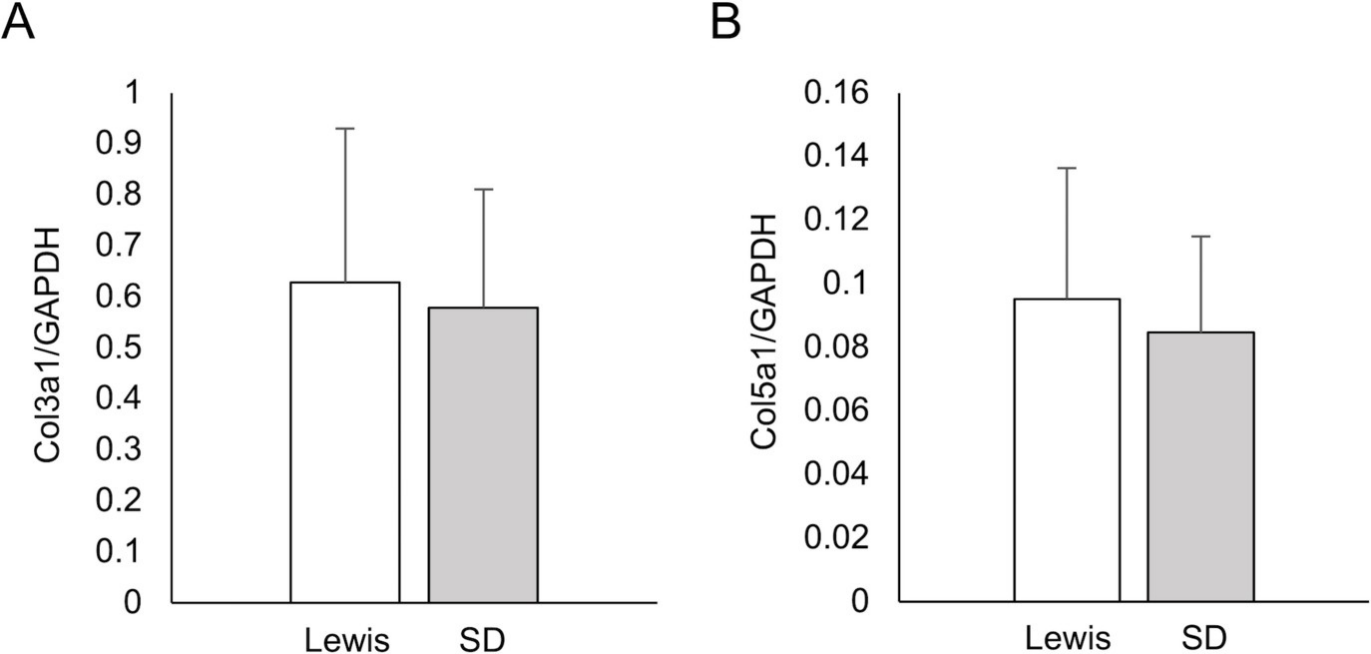
Real-time PCR analyses of the Col3a1 and Col5a1 mRNA expression in the pancreatic tissues of each rat strain. (A) Col3a1/GAPDH ratio. (B) Col5a1/GAPDH ratio. In all analyses, GAPDH was used as a housekeeping gene.

The Col3a1 and Col5a1 mRNA levels in rat pancreatic tissues were similar between the Lewis and SD groups (Col3a1/GAPDH: Lewis 0.628 ± 0.302 vs. SD 0.579 ± 0.231, P = 0.92; Col5a1/GAPDH: Lewis 0.095 ± 0.041 vs. SD 0.085 ± 0.030, P = 0.92) (Fig 4A and 4B).

### Protein expression of collagen III and collagen V in rat pancreatic tissues

**Fig 5:**
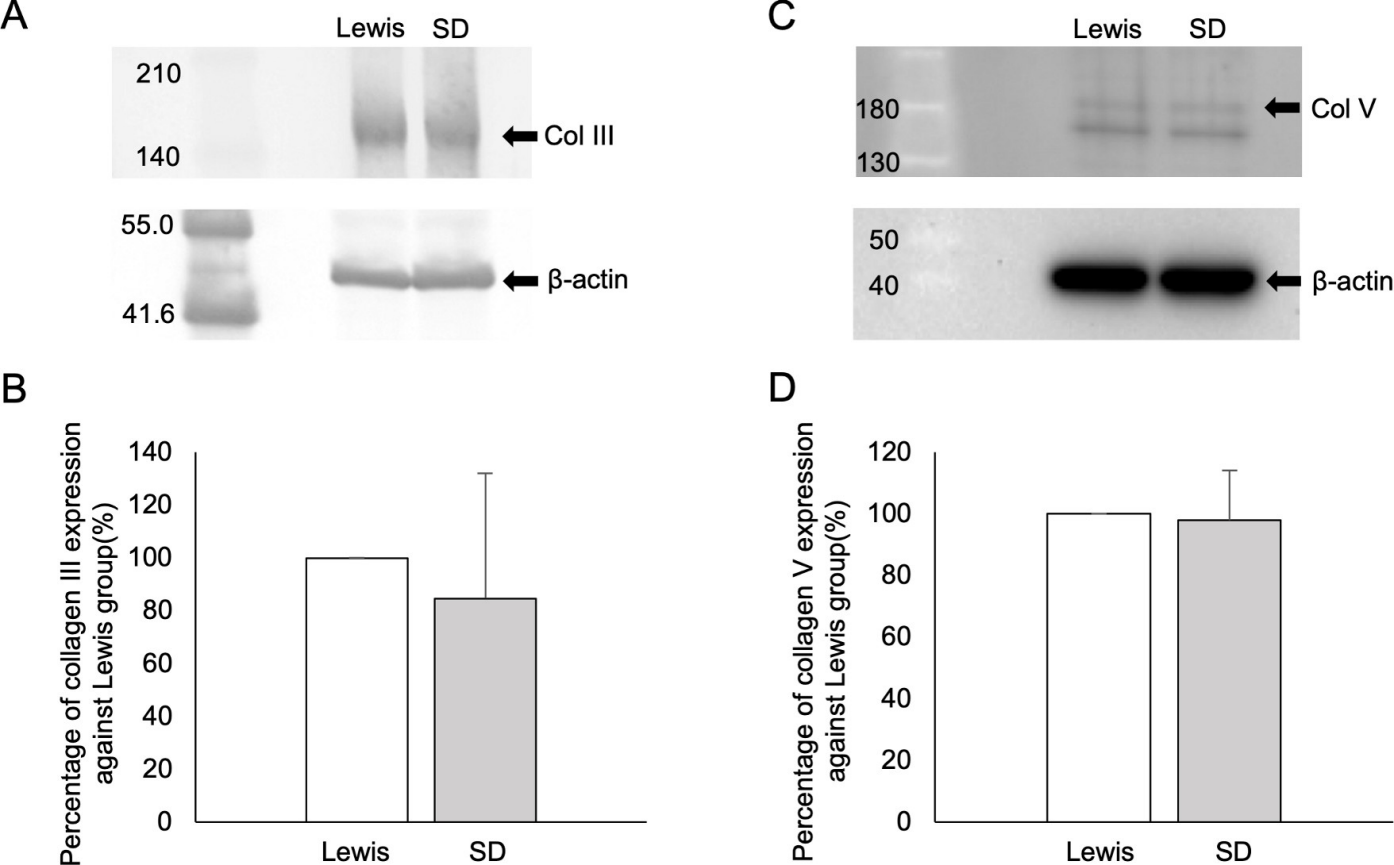
**Western blot analyses of Col III (A, B) and Col V (C, D) in the pancreatic tissues of each rat strain.** The findings for SD rats are presented as the percentage when those of Lewis rats were considered 100% (B, D). β-actin was used as a control (A, C).

The Col III protein expression in the Lewis rat pancreatic tissues was considerably higher than in the SD rat tissues (100.0 ± 0.0 vs. 84.7 ± 47.3, p = 0.10, n = 5) (Fig 5A and 5B). However, no marked difference was detected between the two groups in terms of the Col V expression (Fig 5C and 5D).

### Quantitative analyses of collagens in rat pancreatic tissues by MS

**Fig 6:**
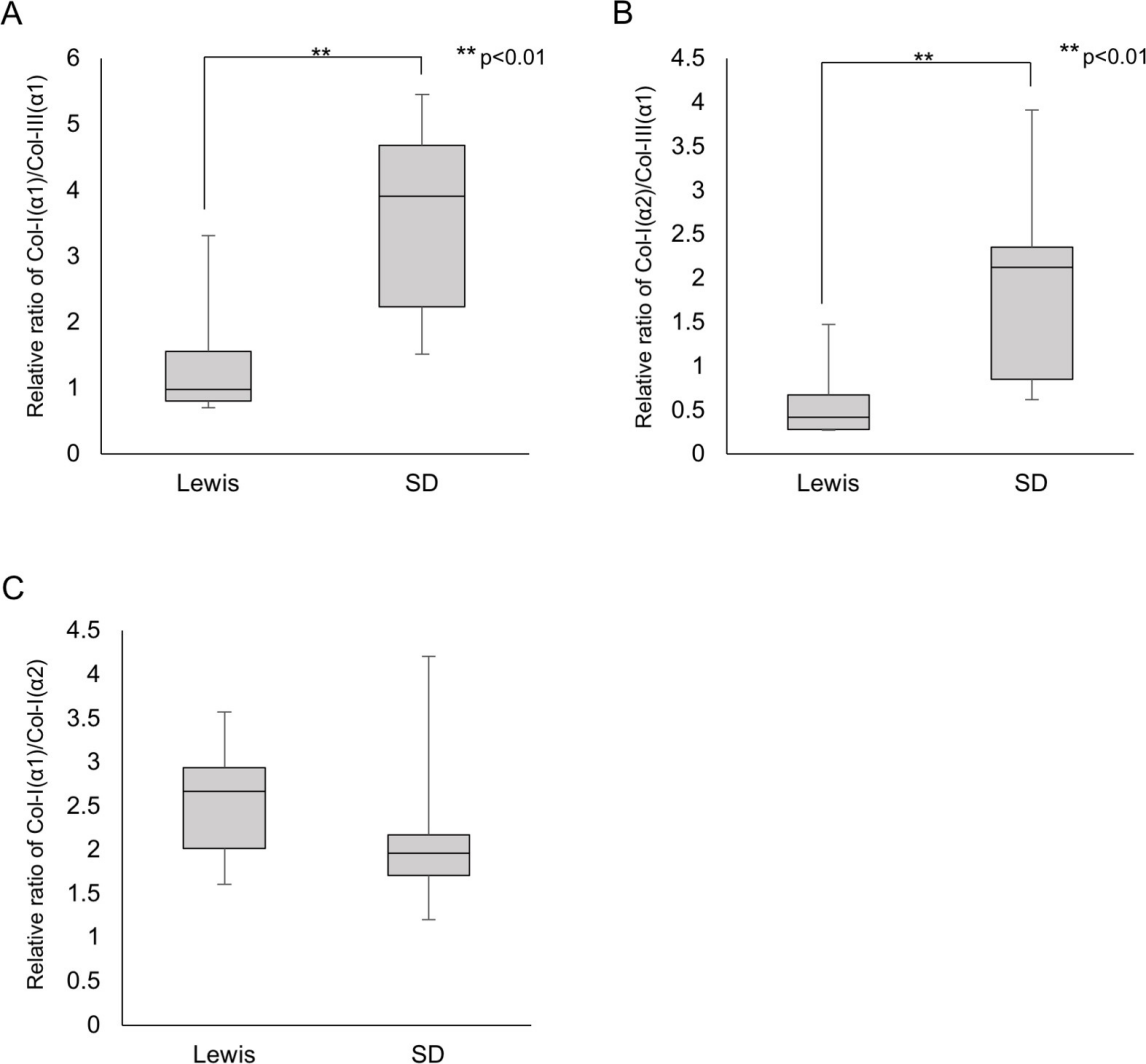
MS Analyses of pancreatic tissues in each rat strain. (A) Col-I(α1)/Col-III(α1) ratio. (B) Col-I(α2)/Col-III(α1) ratio. (C) Col-I(α1)/Col-I(α2) ratio.

In the MS analyses of collagens, the α1 and α2 chains of collagen type I (Col-I(α1) and Col-I(α2)) and the α1 chain of collagen type III (Col-III(α1)) were detected in all samples. Quantitative analyses were conducted for relative values using the ratio Col-I(α1)/Col-III(α1), Col-I(α2)/Col-III(α1) and Col-I(α1)/Col-I(α2). Fig 6 shows the collagen ratios evaluated from the emPAI index. On comparing the Lewis and SD rats, Lewis rats had significantly lower values for Col-I(α1)/Col-III(α1) and Col-I(α2)/Col-III(α1) than SD rats (Col-I(α1)/Col-III(α1): 1.467 ± 1.102 vs. 3.546 ± 1.534, p = 0.007, n = 8; Col-I(α2)/Col-III(α1): 0.605 ± 0.462 vs. 1.907 ± 1.109, p = 0.007, n = 8) (Fig 6A and 6B). These values indicate two possibilities: (1) Col-III(α1) content is Lewis > SD, or (2) Col-I(α1) and Col-I(α2) content are Lewis < SD.

Collagen type I exists as a trimeric form consisting of two α1 chains and one α2 chain (heterotrimer) or three α1 chains (homotrimer) [23]. Assuming a complete heterotrimer, the chain ratio of Col-I(α1)/Col-I(α2) should be two, so a mixture of two forms should show a chain ratio of more than two. Our results indicated that Col I exists as a mixture of the two forms, with a ratio (homotrimer/heterotrimer) of 0.186:1 for Lewis rats and 0.044:1 for SD rats.

## Discussion

To establish a tailor-made donor tissue-specific islet isolation protocol, two factors appear to be essential. The first is a highly purified tissue dissociation enzyme. If poorly characterized unknown proteases derived from *C*. *histolyticum* are included in the dissociation enzyme, the equation between the dissociation enzyme and target matrix will not work efficiently. The second crucial factor is identifying the target extracellular matrix of each enzyme component. We previously reported that Col III is a key target matrix of ColH [13], and Col V is a key substrate for ColG using highly purified recombinant collagenase of each subtype [17]. Based on these novel findings, we investigated the relationship between Col III and Col V expression in pancreatic tissues and influence of ColG:ColH ratio on islet isolation outcome in different rat strain. Our data suggested that the Col III expression and/or the composition of homotrimeric Col I in pancreatic tissues may be useful for optimizing the ColG:ColH ratio in islet isolation.

We observed no beneficial effects by increasing the ColG amount irrespective of the rat strain. This tendency was consistent with previous reports [13, 24]. In contrast, the dependency on the ColH component seemed to be distinct between the two strains. Given that Col III was a key target matrix of ColH in our previous study [13], the Col III expression in Lewis pancreatic tissues may be higher than that in SD rats. A semi-quantitative analysis using MS showed that Lewis rats had significantly lower Col-I/Col-III ratios, thereby suggesting two possibilities: (1) Col-III content is Lewis > SD, and/or (2) Col-I content is Lewis < SD. Given that Western blot analyses also showed that the Col III protein expression in the Lewis rat pancreatic tissues was considerably higher than that in the SD rats, it seems more likely the former is the case in the present study. Given that Col I is known to be broadly and abundantly expressed in most tissues, this interpretation makes sense.

In the MS analyses, we detected relative amounts of Col I for α1 and α2 chains. Almost no studies have explored the difference in the chain types in Col I. Collagen type I includes a heterotrimer and a homotrimer. Of note, the homotrimer is found only in fetal tissue, fibrosis, and cancers, suggesting it is a relatively minor component; however, the structural and mechanical differences have been discussed [25]. Our findings demonstrated considerable difference in the trimer components between the two rat strains, and Lewis rats tended to have homotrimers more often than SD rats. We intend to evaluate the influences of the trimer component differences on collagenase digestion in our next study. However, the present findings suggest that the outcome of islet isolation with various ColG:ColH ratios and the composition of homotrimeric Col I might be correlated. A quantitative investigation of α chain subtypes in each collagen using MS may also be useful for analyzing collagen IV, since it consists of six α chains. This may be an approach to evaluate the quantitative differences among donors.

In our study, the IHC technique was used to assess the expression of Col III and Col V in pancreatic tissues. IHC staining is an established technique for detecting substances using a specific antibody and clarifying their distribution within tissues. Despite the isolation outcome suggesting that Lewis rat pancreas contains more Col III than SD rat pancreas, IHC staining for Col III did not reveal a stronger expression in Lewis rats. This is likely due to the technical characteristics of our IHC evaluation. Since collagen has a triple-helical structure [26, 27] and IHC staining only evaluates from a two-dimensional perspective, IHC staining can only detect the part of the collagen structure that appears on the surface of pancreatic histological sections. As such, target epitopes of the antibody may be inaccessible due to the native three-dimensional conformation in the pancreas. Furthermore, IHC can only assess parts of the whole pancreatic tissues. In support of this hypothesis, the results of Western blotting showed that Lewis rat pancreas contained substantially higher amounts of Col III than SD rat pancreas. This discrepancy between the IHC and Western blot results may be due to the better exposure of the target linear epitopes in the Western blot analysis and less sensitivity on quantification in the IHC.

To further evaluate the expression of Col III and Col V in pancreatic tissues, we assessed Col3a1 and Col5a1 genes using real-time PCR. Of particular interest, the expression of Col5a1 was markedly lower than that of Col3a1, possibly due to the extreme importance of ColH in rat pancreatic islet isolation [13]. The Col3a1 expression was slightly higher in Lewis rats than in SD rats, whereas the expression of Col5a1 had no tendency to correlate with the outcome of islet isolation. The Col3a1 expression tended to correlate with the isolation outcome, but the difference was small and not significant. One possible explanation for the discrepancy between the real-time PCR and Western blot findings may be the involvement of hydroxyproline. In general, collagens include a hydroxyproline to stabilize the collagen triple helix. This hydroxyproline is produced during a post-translational modification process. The hydroxylation of proline is not unified and can occur at any of several positions [28]. Although both the real-time PCR and Western blot assays assessed the total amount of each collagen type, post-translational modification cannot be taken into consideration in real-time PCR. Therefore, the difference between these real-time PCR and Western blot findings might be due at least in part to a significant degree of hydroxylation for proline.

In summary, the present study revealed that an appropriate analysis technique taking the three-dimensional structure of collagen into account is needed in order to accurately evaluate the relative composition of collagen types in pancreatic tissues. Of particular note, the Col III expression and/or the composition of homotrimeric Col I in pancreatic tissues using MS may aid in the optimization of the ColG:ColH ratio. To establish a tailored donor-specific islet isolation approach, further analyses of collagen subtypes in human pancreatic tissues using MS are required.

## Supporting information

S1 Fig Western blot analysis of Col III. (TIF)

S2 Fig Western blot analysis of β-actin (control of Col III analysis). (TIF)

S3 Fig Western blot analysis of Col V. (TIF)

S4 Fig Western blot analysis of β-actin (control of Col V analysis). (TIF)

S1 Table Detailed results of the present study. (XLSX)
