# Supplementary figures and images for: Strategy towards tailored donor tissue-specific pancreatic islet isolation
Source: PLoS One. 2019 May 10;14(5):e0216136. doi: 10.1371/journal.pone.0216136 (PMC6510438; doi:10.1371/journal.pone.0216136)

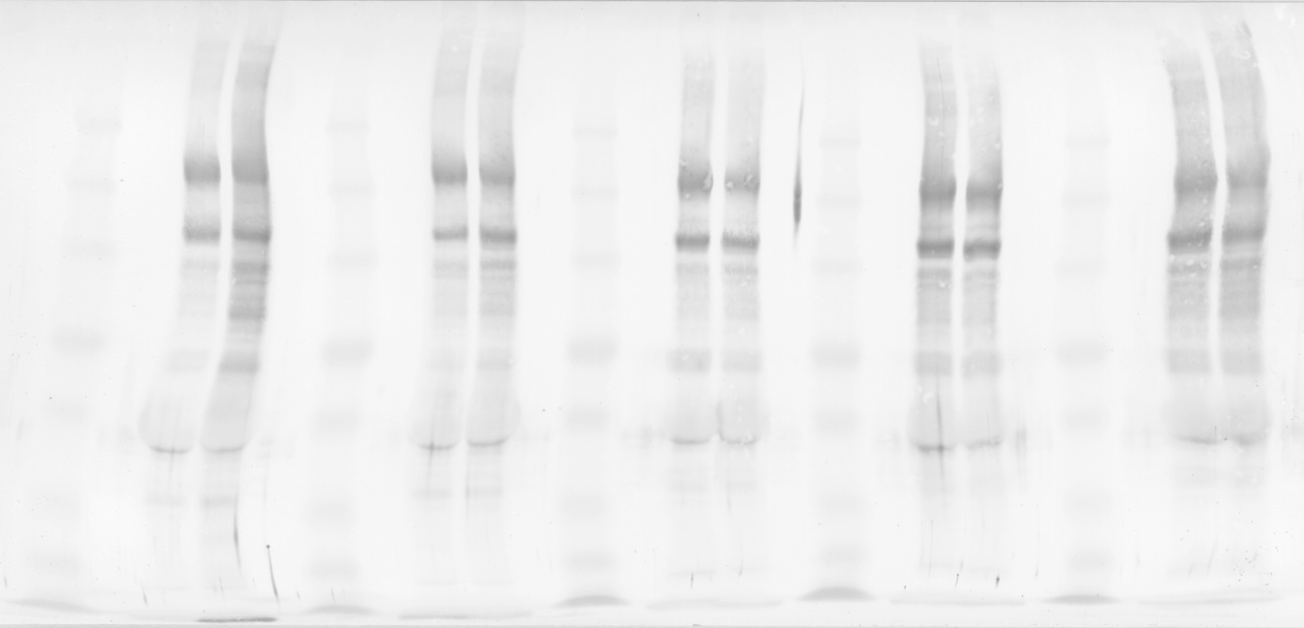

Supplement: S1 Fig — (TIF) [file pone.0216136.s001.tif]

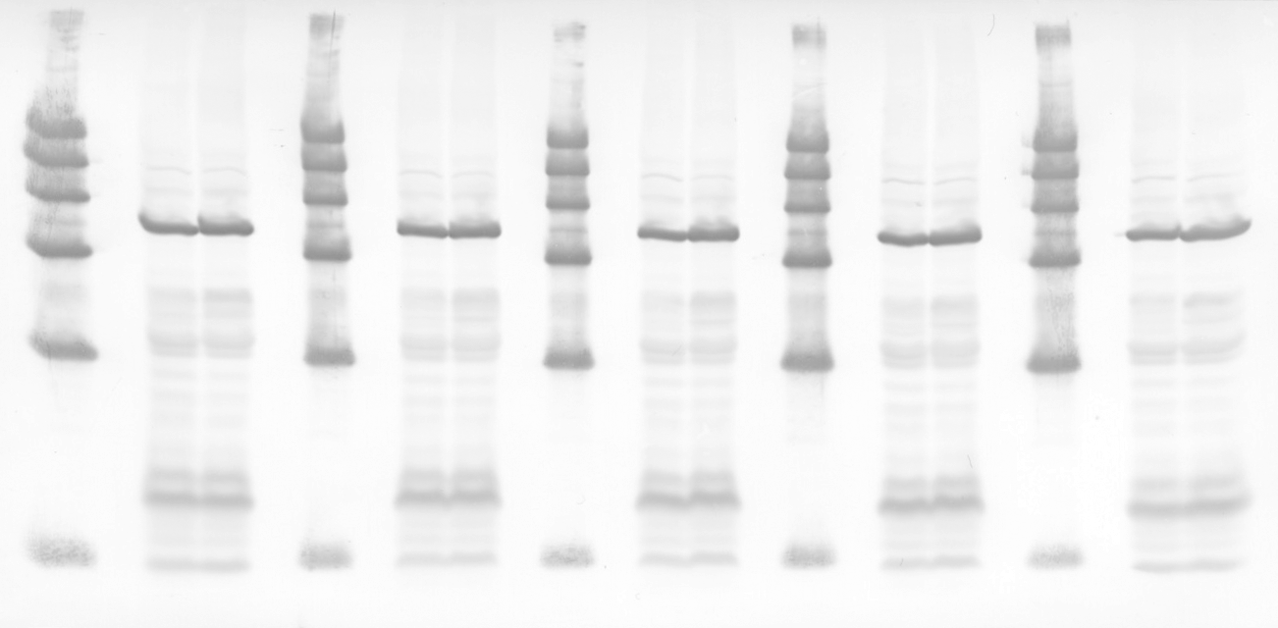

Supplement: S2 Fig — (TIF) [file pone.0216136.s002.tif]

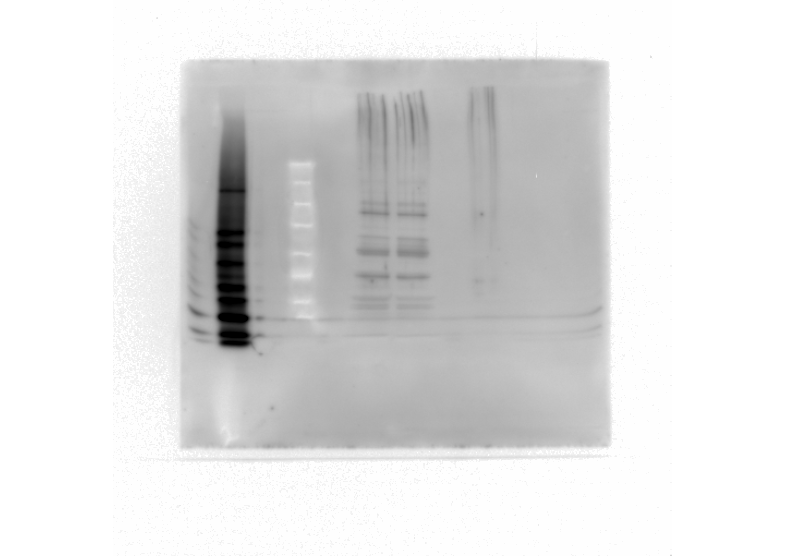

Supplement: S3 Fig — (TIF) [file pone.0216136.s003.tif]

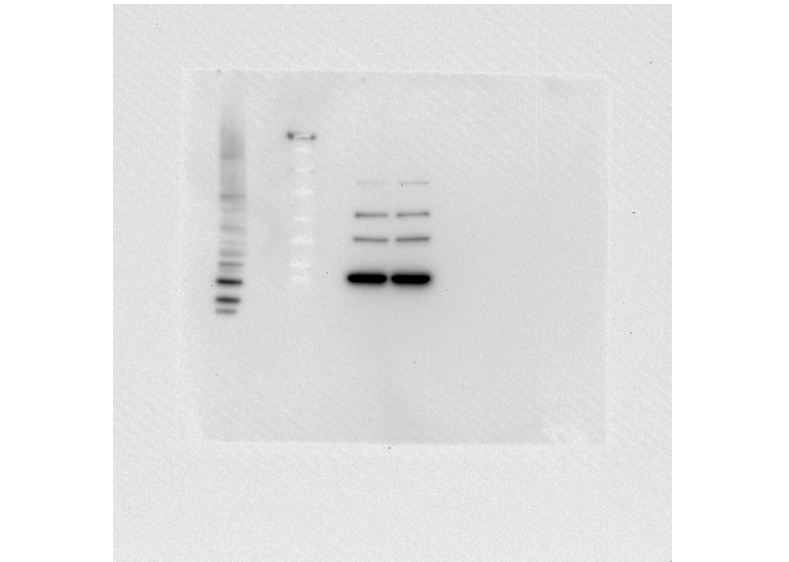

Supplement: S4 Fig — (TIF) [file pone.0216136.s004.tif]
